# Improved Acquisition and Reconstruction for Wavelength-Resolved Neutron Tomography †

**DOI:** 10.3390/jimaging7010010

**Published:** 2021-01-15

**Authors:** Singanallur Venkatakrishnan, Yuxuan Zhang, Luc Dessieux, Christina Hoffmann, Philip Bingham, Hassina Bilheux

**Affiliations:** 1 Multimodal Sensor Analytics Group, Oak Ridge National Lab, Oak Ridge, TN 37831, USA; binghampr@ornl.gov; 2 Neutron Scattering Division, Oak Ridge National Lab, Oak Ridge, TN 37831, USA; zhangy6@ornl.gov (Y.Z.); dessieuxll@ornl.gov (L.D.); choffmann@ornl.gov (C.H.); bilheuxhn@ornl.gov (H.B.)

**Keywords:** model-based imaging, hyperspectral tomography, interlaced scanning, streaming tomography

## Abstract

Wavelength-resolved neutron tomography (WRNT) is an emerging technique for characterizing samples relevant to the materials sciences in 3D. WRNT studies can be carried out at beam lines in spallation neutron or reactor-based user facilities. Because of the limited availability of experimental time, potential imperfections in the neutron source, or constraints placed on the acquisition time by the type of sample, the data can be extremely noisy resulting in tomographic reconstructions with significant artifacts when standard reconstruction algorithms are used. Furthermore, making a full tomographic measurement even with a low signal-to-noise ratio can take several days, resulting in a long wait time before the user can receive feedback from the experiment when traditional acquisition protocols are used. In this paper, we propose an interlaced scanning technique and combine it with a model-based image reconstruction algorithm to produce high-quality WRNT reconstructions concurrent with the measurements being made. The interlaced scan is designed to acquire data so that successive measurements are more diverse in contrast to typical sequential scanning protocols. The model-based reconstruction algorithm combines a data-fidelity term with a regularization term to formulate the wavelength-resolved reconstruction as minimizing a high-dimensional cost-function. Using an experimental dataset of a magnetite sample acquired over a span of about two days, we demonstrate that our technique can produce high-quality reconstructions even during the experiment compared to traditional acquisition and reconstruction techniques. In summary, the combination of the proposed acquisition strategy with an advanced reconstruction algorithm provides a novel guideline for designing WRNT systems at user facilities.

## 1. Introduction

**Figure 1:**
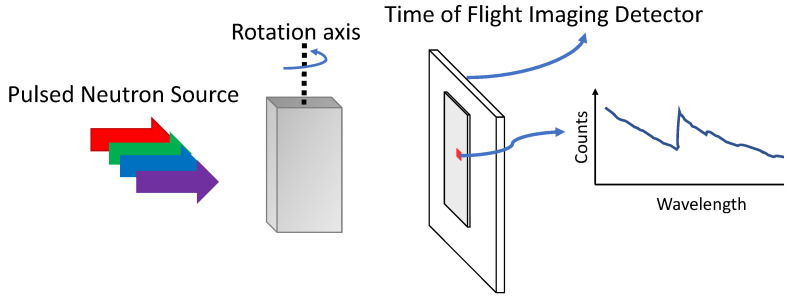
A block diagram of the wavelength-resolved neutron tomography setup at a spallation neutron source. An incident hyper-spectral neutron beam, depicted here with multiple color arrows each corresponding to a neutron wavelength, provides the transmission signal through a sample placed in front of the detector. A time-of-flight (TOF) imaging detector measures the number of neutrons detected as a function of time, which is equivalent to wavelength. These measurements can be binned into wavelengths to obtain a projection measurement corresponding to each wavelength and rotation angle.

WRNT is a technique used to characterize samples in 3D based on the time-of-flight (TOF) technique that is capable of identifying the time of arrival of each neutron, thus its wavelength. Each WRNT scan involves collecting WR projection data of an object from multiple orientations and then using an algorithm to obtain a hyper-spectral 3D reconstruction. Figure 1 shows a schematic of a WRNT instrument where a spectral source of neutrons, typically produced by the spallation of neutrons from the interaction of a proton beam with a heavy element target such as tungsten or mercury, impinges on the sample of interest which is then rotated to obtain a full set of tomographic measurements. WRNT is performed at spallation neutron sources based facilities [1,2,3,4] by using a pulsed (spectral) source combined with a time-of-flight detector [5] that allows events of different wavelengths, which are inversely proportional to their kinetic energy, to be recorded according to arrival time at the detector. Wavelength-resolved neutron imaging has been used to study the phase composition of samples [6,7] and to investigate the crystalline nature of engineering materials [8] using the principles of Bragg scatter i.e., making use of the fact that crystals diffract strongly when they satisfy the Bragg condition at a certain orientation and neutron wavelength [9].

While WRNT beam lines are in operation, there are challenges that still have to be addressed in order to produce high-fidelity tomographic reconstructions at these facilities. Specifically, the first phase of innovations were mainly focused on the instrument design for producing the highest flux possible, and efficient detectors to get high-quality measurements. As a result, there has been relatively less focus on the joint design of acquisition and reconstruction algorithms to further improve the performance of the tomographic system. A typical approach for the tomography involves rotating the sample sequentially in a $180^{\circ }$ or $360^{\circ }$ range and obtaining a dataset corresponding to hundreds of wavelength-resolved projection measurements at each position (see Figure 1)—a sequential step-and-scan approach. The data from all the measurements are then processed at the end of the experiment using an analytic reconstruction algorithm such as filtered back projection (FBP) to obtain a tomographic reconstruction [7,10]. However, this approach has a few drawbacks. Firstly, the measured data can be of very low signal-to-noise (SNR) in each wavelength bin due to the limited time available for an experiment. Next, the limited availability of beam time at facilities often forces users to acquire a sparse set of projection data. This results in a reconstruction with significant artifacts when FBP-like algorithms are used for this sparse-view and noisy dataset. Finally, the time taken to acquire a single tomographic scan can be long with experiments spanning several days during which the user may not have accurate feedback on the current state of the reconstruction from the thus far acquired data. In summary, while there has been significant improvements in WRNT systems, most of these have focused on the hardware, leaving open the possibility of further improvements via the design of new acquisition and reconstruction algorithms.

In this paper, we propose a new framework for WRNT acquisition and reconstruction based on the use of an interlaced scanning strategy combined with a model-based image reconstruction algorithm. A key benefit of our approach is that we can produce intermediate reconstructions which significantly suppress noise and artifacts compared to traditional approaches. This also implies that the final reconstructions are available at the end of the experiment time and are of higher quality (lower noise for a given resolution) compared to the FBP methods in [7,10]. Specifically, we adapt the scanning technique of the time-interlaced model-based iterative reconstruction (TIMBIR) algorithm [11] designed in the context of time-resolved synchrotron based X-ray tomography in order to improve the spatio-temporal resolution. We note that an approach which is intuitively similar has also been explored in the context of white-beam time-resolved neutron tomography where a acquisition based on the golden ratio [12] has been used to improve the spatio-temporal resolution of the reconstructions [13,14,15]. While the two acquisition schemes are similar in spirit, the interlaced scanning algorithm of [11] is more general than the golden section method and can be made to be very similar to the golden section method by setting the appropriate parameters during acquisition. In contrast to the sequential step-and-scan approach, the interlaced scan ensures that subsequent measurements are further apart so that there is sufficient diversity in the data at any given time during data acquisition. A benefit of such a strategy is that if there are unexpected downtimes in the course of an experiment, it allows for a more uniform set of measurements to be available for reconstruction compared to traditional sequential scanning protocols. Secondly, we propose to use of a model-based image reconstruction (MBIR) algorithm on the acquired wavelength-resolved/hyper-spectral data instead of the standard FBP techniques. The MBIR algorithm works by minimizing a cost function that balances a data-fitting term that incorporates a physics based model for the imaging system and noise characteristics of the detector, and a regularization term that incorporates a model for the underlying 3D object itself (such as local smoothness). MBIR methods have enabled significant improvements in performance for several tomography applications including for neutron tomography [16,17,18,19] especially when dealing with sparse and low signal-to-noise ratio (SNR) data; a scenario that is also common in WRNT as we seek to provide high-quality real-time feedback to users in the course of an experiment. We demonstrate the utility of our proposed method by implementing this system at the SNAP beam line of the Spallation Neutron Source (SNS) at the Oak Ridge National Laboratory (ORNL) and demonstrate real-time feedback capability during the course of a WRNT of a magnetite sample.

The rest of this paper is organized as follows. In Section 2 we present specific details of the proposed acquisition algorithm and the MBIR method. In Section 3 we provide experimental results from the measurement of a magnetite sample highlighting the utility of the proposed method for WRNT. Finally, in Section 4 we present our conclusions and discuss future research directions.

## 2. Interlaced Scanning and Model-Based Image Reconstruction

Our objective in the design of WRNT systems is to be able to make measurements such that we can provide real-time feedback to users via intermediate high-quality reconstructions and also ensure the same high-quality reconstruction upon completion of the experiment. We achieve these objectives via the design of a new acquisition protocol and combine it with a MBIR algorithm that can produce high-quality reconstructions from sparse, noisy and in-complete data sets.

### 2.1. Interlaced Scanning

**Figure 2:**
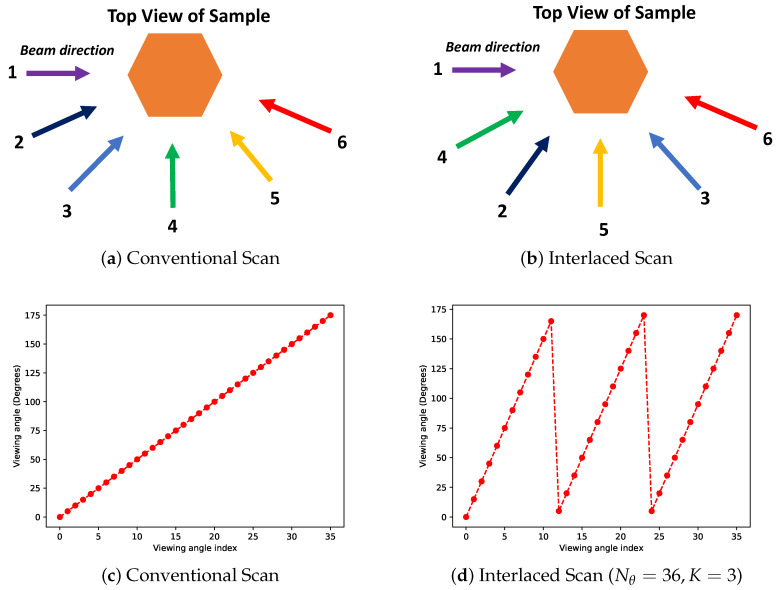
Illustration of two ways to acquire tomographic datasets. (**a**) Representation of the conventional scanning protocol using 6 equally spaced angles between $0^{\circ }$ and $180^{\circ }$. (**b**) Example of one realization of the proposed interlaced scanning protocol that ensures subsequent measurements are far apart ($K=2,N_{\theta }=6$ in Equation (1)). List of view angles using (**c**) the conventional approach and (**d**) one realization of the interlaced scan that was used in the experiments in this paper ($K=3,N_{\theta }=36$). The advantage of the interlaced scan is that at any given point during the course of the experiment, the object is scanned fairly uniformly, thereby enabling reconstructions with fewer artifacts that the end users can utilize to make decisions. The parameters of the interlaced scan can be further adjusted so that subsequent measurements are maximally separated (not shown here).

The conventional approach to a tomographic scan at a WRNT beam line is to rotate the sample about a single axis, and at each position measure a wavelength-resolved projection dataset. Typically, the sample is rotated using a fixed step size which is chosen based on the total number of measurements to be made in the course of the experiment. The sequence of angles usually follows a linear increasing pattern—starting from $0^{\circ }$ and progressing to $180^{\circ }$ using a fixed step size about the axis of rotation (see Figure 2a). However, the disadvantage of such a scanning protocol is that at any given intermediate time in the course of the scan, the object is only viewed from a very limited angular range; making the reconstruction obtained from such partial data to have significant streak artifacts. This can also lead to challenges if, due to some unforeseen circumstance, beam time is lost during an experiment thus producing a dataset which is heavily sampled only from a small angular range. In order to address this challenge, we require that the measurements are spread apart and process the sparse set of measurements using appropriate algorithms so that we can provide a continuous high-quality reconstruction to the end users (see Figure 2b).

2

Here, we propose to use an interlaced scanning strategy [11] to acquire the data. Based on the time available for a given scan (with a reasonable SNR), we can decide on the total number of measurements we plan to measure ($N_{\theta }$). Once $N_{\theta }$ is determined, the conventional data acquisition generates a uniform set of $N_{\theta }$ measurements between $0^{\circ }$ and $180^{\circ }$ (see Figure 2a,c). In contrast to the conventional data acquisition approach described above, we propose to use the method described in [11] which utilizes a sequence of angles that are interlaced so that subsequent angles are far apart. In this algorithm, in addition to $N_{\theta }$, we choose a number, *K*, which represents the number of “half-rotations” over which to acquire the data. This *K* ($\leq N_{\theta }$) can be chosen by the user who desires to obtain roughly *K* reconstructions in the course of an experiment (though other heuristics can be also used). Once these numbers are chosen, the list of angles, $\theta_{n}$, (in the range $0^{\circ }$ to $180^{\circ }$) is given by

(1) $$\theta_{n}=\left[\left(n\;\;\mathrm{mod}\;\frac{N_{\theta }}{K}\right)K+B_{r}\left(\frac{nK}{N_{\theta }}\;\;\mathrm{mod}\;K\right)\right]\frac{180}{N_{\theta }}$$

$\forall n\in \left\{0,..,N_{\theta }-1\right\}$, mod is the standard modulo operator, and $B_{r}$ is the bit-reversal operator that takes the integer representation of a number and reverses the order of the bits [20]. An example of this sequence for N=36 and K=3 is shown in Figure 2d and is contrasted with the standard sequential scanning protocol in Figure 2c. Notice that both the sampling methods acquire the same set of views, but the main difference is in the order in which these views are measured. When $K=N_{\theta }$ the resulting sequence will be maximally separated i.e., each subsequent measurement is farthest apart from the previous, which is in complete contrast to the conventional scanning where each subsequent measurement is closest to the previous one for a given $N_{\theta }$. Hence, at the end of the experiment, both protocols will have measured the same set of views but the interlaced scan acquires data in a manner that covers the full angular range better even at intermediate times in the experiment. The advantage of interlaced scanning is that if there is a disruption, say mid-way through an experiment, we can obtain a reconstruction with fewer artifacts than from a conventional scan. Furthermore, if we desire to provide intermediate reconstructions to the end-users during an experiment, this scanning protocol can enable higher-quality reconstructions for a generic sample compared to the traditional scan as we will demonstrate in the results section. We note that such sampling patterns are best used when the time to rotate the object from one position to the next is smaller than the time to make the measurement itself, which is typical for WRNT systems where it can take of the order of an hour or more to acquire one projection measurement compared to a few seconds to align the sample in a particular direction with respect to the beam. Finally, we note that for this case the interlaced scanning strategy is similar but not exactly the same as the golden ratio based scan of [13] that has been investigated for time-resolved neutron CT. One of the advantages of the proposed strategy compared to the golden section method is that the parameter *K* can be adjusted to control how separated successive angles are.

### 2.2. Model-Based Image Reconstruction for Streaming Hyper-Spectral Data

Model-based image reconstruction [21] approaches have been developed for the last few decades and are well established to be the method of choice when dealing with sparse, limited-view and noisy tomographic datasets. In the scientific user facility community, such methods have been developed for electron tomography [22,23,24], synchrotron based X-ray CT [11,25,26] and neutron laminography and tomography [16,17,18,19]. In this section, we briefly explain the core ideas behind MBIR and how we have adapted it for the WRNT systems in order to perform hyper-spectral tomography. We skip some of the mathematical details since these are well established in the literature.

At any given stage in the course of the experiment, when *n* views have been measured, the MBIR is obtained as

(2) $$\begin{array}{c} {\hat{f}}^{\left(n\right)}\leftarrow \underset{f}{\mathrm{argmin}}c^{\left(n\right)}\left(f\right) \end{array}$$

where

(3) $$\begin{array}{c} c^{\left(n\right)}\left(f\right)=\frac{1}{2}\sum_{t=1}^{n}{∥g^{\left(t\right)}-{\bar{A}}^{\left(t\right)}f∥}_{W^{\left(t\right)}}^{2}+\bar{R}\left(f;\psi \right) \end{array}$$

where $g^{\left(t\right)}$ is the vector of normalized hyper-spectral projection measurements at view *t* organized as $g=\left[\begin{array}{c} g_{1}^{\left(t\right)}\\ \vdots \\ g_{S}^{\left(t\right)} \end{array}\right]$, $g_{s}^{t}$ is a vector of all normalized measurements at view *t* and wavelength bin *s*, *f* is the vector containing all the unknown voxels of hyper-spectral attenuation coefficients organized as $f=\left[\begin{array}{c} f_{1}\\ \vdots \\ f_{S} \end{array}\right]$, ${\bar{A}}^{\left(t\right)}$ is a matrix that represents that forward-projection operator at view *t* at all the wavelengths (a block diagonal matrix with entries $A^{\left(t\right)}$ representing the forward projection at view angle $\theta_{t-1}$), $W^{\left(t\right)}$ is a diagonal weight matrix containing the inverse noise-variance of the measurements $g^{\left(t\right)}$, and $\bar{R}\left(f;\psi \right)$ is a regularization term that enforces certain desirable properties in the reconstructed volume (like local smoothness, sharp edges etc.). Notice that this is a very high-dimensional optimization problem over all unknown voxels across all wavelength bins. The entries of the weight matrix ($W^{\left(t\right)}$) are typically set to be the measured (un-normalized) count data in the case of transmission tomography [27] and have the intuition that if this number is very small, the overall term weights less relative to other terms in the cost-function. The inclusion of this term allows for a dose-weighting of the acquired data and can be a useful way to reject some of the individual measurements. In summary, the MBIR approach involves formulating the tomographic reconstruction as minimizing a function that balances a fitting term that enforces a low discrepancy between the projection of the final reconstruction and the true measurements; and a regularization term that enforces certain desirable properties in the reconstruction.

There are many potential choices for the regularization term $\bar{R}$ in Equation (3); some of which can enforce desirable properties in each wavelength bin and others that can model the correlations across bins (i.e., adjacent bins will be correlated). In this paper, we choose an $\bar{R}$ which regularizes each wavelength bin independently.

(4) $$\begin{array}{c} \bar{R}\left(f;\psi \right)=\sum_{s=1}^{S}R\left(f_{s};\psi_{s}\right) \end{array}$$

where $f_{s}$ is a vectorized version of all the voxels in the *s*th wavelength bin, and $\psi_{s}$ is the set of parameters associated with that bin. For R(;) we choose a q-generalized Markov-random field (qGGMRF)-based function [28]. It is given by

(5) $$\begin{array}{ccc} R(x;\psi_{s}) & = & \sum_{\{j,k\}\in N}w_{jk}\rho \left(x_{j}-x_{k};\psi_{s}\right)\\ \rho (x_{j}-x_{k};\psi_{s}) & = & \frac{{\left|\frac{x_{j}-x_{k}}{\sigma_{x,s}}\right|}^{2}}{c+{\left|\frac{x_{j}-x_{k}}{\sigma_{x,s}}\right|}^{2-p_{s}}} \end{array}$$

where $\psi_{s}=\left[p_{s},\sigma_{x,s},c\right]$ are the parameters of the function, N is the set of pairs of neighboring voxels (e.g., a 26 point neighborhood), 1≤p≤2, *c* and $\sigma_{x}$ are qGGMRF parameters. *c* is typically set to a very small number and is used to make the overall function differentiable. The weights, $w_{jk}$, are inversely proportional to the distance between voxels *j* and *k*, normalized to 1. This model provides a greater degree of flexibility in the quality of reconstructions compared to an algorithm specifically designed for a total-variation regularizer that may force the reconstructions to appear “waxy” [21]. In particular, when p=1 we get a behavior similar to a total-variation model and when p=2 the regularizer is a quadratic function allowing for smoother reconstructions. Finally, we note that the choice of regularizer can be significantly improved further compared to the above choice. Exploiting the correlations across wavelength channels in the MBIR framework can help further improve the reconstruction quality as has been observed in several hyper-spectral imaging applications [25,29,30,31]. Furthermore, if we are measuring samples with known materials and spectral responses, this can be used as a part of the model in the MBIR framework to reduce the dimensionality of the problem dramatically (instead of reconstructing every voxel at each channel we only need to find the proportion of each material at each voxel) [31]. However, in this proof-of-concept study we implement the regularizer (5) for its simplicity and the fact that it enables parallel reconstruction of each of the individual hyper-spectral bins.

With these choices of functions, the cost-function (3) can be re-written as

(6) $$\begin{array}{c} c^{\left(n\right)}\left(f\right)=\frac{1}{2}\sum_{t=1}^{n}\sum_{s=1}^{S}{∥g_{s}^{\left(t\right)}-A^{\left(t\right)}f_{s}∥}_{W_{s}^{\left(t\right)}}+\sum_{s=1}^{S}R\left(f_{s};\psi_{s}\right). \end{array}$$

Because we have chosen a regularizer that is separable, this can be re-written as

(7) $$\begin{array}{c} c^{\left(n\right)}\left(f\right)=\sum_{s=1}^{S}\left\{\frac{1}{2}\sum_{t=1}^{n}∥g_{s}^{\left(t\right)}-A^{\left(t\right)}f_{s}∥+R\left(f_{s};\psi_{s}\right)\right\} \end{array}$$

Therefore the hyper-spectral reconstruction after measuring *n* views can be computed as

(8) $$\begin{array}{c} {\hat{f}}_{s}^{\left(n\right)}\leftarrow \underset{f_{s}}{\mathrm{argmin}}\left\{\frac{1}{2}\sum_{t=1}^{n}∥g_{s}^{\left(t\right)}-A^{\left(t\right)}f_{s}∥+R\left(f_{s};\psi_{s}\right)\right\} \end{array}$$

∀s∈1,...,S. Finally, we note that even though

$$c^{\left(n\right)}\left(f\right)=c^{\left(n-1\right)}\left(f\right)+\frac{1}{2}{∥g^{\left(n\right)}-{\bar{A}}^{\left(n\right)}f∥}_{W^{\left(n\right)}}^{2}$$

∀n≥1, the MBIR method involves processing all the data in order to obtain a reconstruction. However, we can attain an accelerated convergence to the solution by initializing the optimization routine at the *n*th stage with the solution from the (n−1)th stage. In contrast to this, linear methods used for tomography like the filtered-back projection can be implemented by keeping track of the current reconstruction and simply adding the contribution from the new measurement.

We use the optimized gradient method (OGM) [32] to find a minimum of the above cost function for each wavelength channel in equation (8). The algorithm involves a standard gradient computation combined with a step size determined using Nesterov’s method. In order to prevent the notation from getting cumbersome, we drop the indices corresponding to the wavelength channel (*s*) and the view index (*t*) and merely highlight what forms the core of each iteration. Specifically, for each iteration *k*,

(9) $$\begin{array}{ccc} h^{\left(k+1\right)} & \leftarrow & x^{\left(k\right)}-\frac{1}{L}\nabla c\left(x^{\left(k\right)}\right) \end{array}$$

(10) $$\begin{array}{ccc} t^{\left(k+1\right)} & \leftarrow & \frac{1+\sqrt{1+4{\left(t^{\left(k\right)}\right)}^{2}}}{2} \end{array}$$

(11) $$\begin{array}{ccc} x^{\left(k+1\right)} & \leftarrow & h^{\left(k+1\right)}+\frac{t^{\left(k\right)}-1}{t^{\left(k+1\right)}}\left(h^{\left(k+1\right)}-h^{\left(k\right)}\right)+\frac{t^{\left(k\right)}}{t^{\left(k+1\right)}}\left(h^{\left(k+1\right)}-x^{\left(k\right)}\right) \end{array}$$

where $t^{\left(0\right)}=1$, *L* is the Lipschitz constant of the gradient of c(.), $h^{\left(0\right)}=x^{\left(0\right)}$ is an initial estimate for the reconstruction. The gradient of the cost-function c(.) is given by

(12) $$\begin{array}{c} \nabla c\left(x\right)=-A^{T}W\left(g-Ax\right)+\nabla R\left(x;\psi \right). \end{array}$$

We use the ASTRA toolbox [33,34] to implement GPU accelerated forward (*A*) and back-projection ($A^{T}$) operators. We also accelerate the computation of the expression for ∇R(x;ψ) using a parallel implementation. Overall, this leads to a algorithm which can be implemented rapidly for each wavelength channel and across all channels in parallel.

Our overall framework for acquisition and reconstruction is summarized in Algorithm 1. We note that it is not necessary to reconstruct the data in order to proceed to the next measurement in the proposed framework. Furthermore, MBIR methods are computationally expensive and obtaining real-time reconstructions depends on the acquisition time, size of the detectors and the number of wavelength bins. In practice, for the type of samples and detectors used in this paper we have observed that it is possible to obtain a useful (partial) reconstruction from the data in lesser time than it takes to measure the next projection using our implementation of the algorithm. We believe that using even more optimized implementations [35,36] we can obtain large hyper-spectral reconstructions in near-real time for WRNT systems using fairly small compute clusters. We plan to open-source our reconstruction code as a part of the pyMBIR package [37] upon publication of the current work.

**Algorithm 1:** The proposed framework for wavelength-resolved neutron tomography systems. Prior to the scan we decide a fixed number of views to measure $N_{\theta }$ and the number of half-rotations over which this will occur *K*. After each measurement the object can be reconstructed using the MBIR algorithm. The reconstruction from the previous stage is used as a initial condition for the next stage in order to accelerate the convergence i.e., time to reconstruct for the MBIR method.1: **function**$[{\hat{f}}^{\left(N_{\theta }\right)}]\leftarrow$ Reconstruct($N_{\theta }$,*K*,ψ)2:    %**Inputs**: Number of views $N_{\theta }$, Number of half-rotations *K*, Regularization parameters ψ
3:    %**Outputs**: Wavelength-resolved reconstruction ${\hat{f}}^{\left(N_{\theta }\right)}$
4:    Compute $\theta_{n}$ according to (1) $\forall n\in \left\{0,..,N_{\theta }-1\right\}$
▹ Interlaced scanning angles
5:    Initialize ${\hat{f}}^{\left(0\right)}\leftarrow 0$6:    Measure open-beam data $\lambda_{o,s}\forall s\in \left\{1,..,S\right\}$▹ Normalization data for transmission tomography7:    n←18:    **while**
$n\leq N_{\theta }$
**do**9:        $\lambda^{\left(n\right)}\leftarrow$ MEASURE${}_{PROJECTION}$($\theta_{n-1}$)10:        $g^{\left(n\right)}\leftarrow$ NORMALIZE($\lambda^{\left(n\right)}$,$\lambda_{o}$)▹ Can include further corrections11:        ${\hat{f}}^{\left(n\right)}\leftarrow$ MBIR ($g^{\left(1\right)},..,g^{\left(n\right)}$; ψ,${\hat{f}}^{\left(n-1\right)}$)▹ Equation (8)12:        n←n+113:    **end while**14:    Return ${\hat{f}}^{\left(N_{\theta }\right)}$15: **end function**

## 3. Results

**Figure 3:**
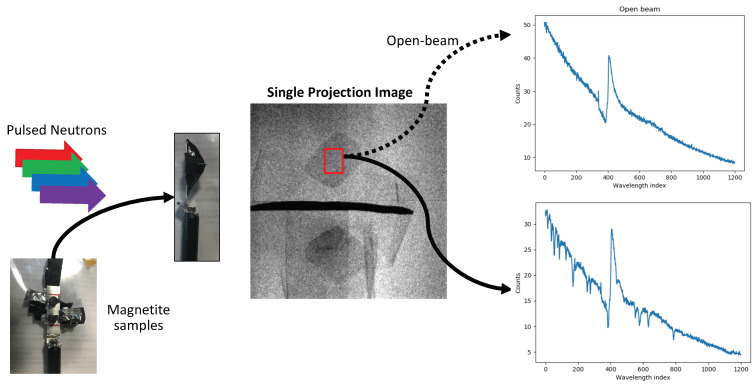
Illustration of the experimental set-up. Two magnetite crystals are mounted on a holder, separated by a piece of cadmium and held by a tape. Each projection image consisted of measurements using a 512×512 micro-channel plate detector which natively binned the images into energy-bins. Each such projection measurement took approximately 1.5 h of experiment time and the counts per bin in a region of the sample were approximately in the range of 0 to 35 counts as shown in the plot above (average signal strength in a region indicated by the red box).

In order to illustrate our approach, we acquired a dataset consisting of two magnetite samples (see Figure 3) using the SNAP instrument at the Spallation Neutron Source at Oak Ridge National Laboratory. The samples were taped to a Aluminum post and separated by a piece of Cadmium. The acquisition time for each projection image was set so that the accumulated proton charge was set to 4 Coulombs and a 512×512 pixel micro-channel plate detector (MCP) [5] was used to acquire the data. The acquisition hardware was programmed in order to acquire an interlaced scanning pattern using K=3 and N=36 (see Figure 2). We note that due to a change in beam power, the acquisitions took longer than anticipated and we only acquired 30 projection measurements in the course of the allotted beam time. Furthermore, for the exposure time we had chosen (approximately 1.5 h corresponding to the accumulated charge of 4 Coulombs) the signal-to-noise ratio of the data was low (see Figure 3) with counts in the range of approximately 10 to 50 in a region of the sample in each wavelength bin. For each projection measurement, we binned the data into a total of 1400 time-of-flight/wavelength bins and used the first 1200 bins for the reconstructions. As the data was acquired, we pre-processed it using a median filter with a window size of 3 to reduce the impact of outliers, combined four adjacent wavelength bins by summing them in order to boost the SNR, normalized the data by applying the log operation to the ratio of the open-beam measurements to the acquired measurements with the sample, and then applied a stripe removal filter (Fourier wavelet algorithm) using TomoPy [38] to suppress ring-artifacts in the reconstruction. While we accumulated the counts in the four adjacent bins for these results, in the future we anticipate being able to do so during acquisition i.e., choosing the size of the wavelength/time-of-flight bins so that the acquired data has a sufficient signal-to-noise ratio and satisfies the wavelength resolution needed for the specific scientific use case. We then reconstructed the resulting normalized and binned data using the FBP and MBIR methods and compared the reconstructions obtained using two different scanning protocols— (1) The conventional method where we sequentially measure the sample from 0 to 180 degrees in the course of an experiment and (2) The interlaced scanning protocol proposed in this work. We note that since we only performed the experiment once using the interlaced scanning protocol, the results from the conventional scanning protocol are obtained by collecting all the data and retrospectively processing it as if it were acquired using the conventional method. The filter cut-off parameter for FBP was set to 0.35 for all wavelength bins and the Ram-Lak filter was used. The $p_{s}$ was set to 1.2 for MBIR and the $\sigma_{x,s}$ was set to 0.4 for all the wavelength-bins. These parameter values produced reconstructions of reasonable visual quality i.e., a reasonable balance between noise levels and visual resolution. The question of how to choose the parameters is an open question for WRNT with our observation that this is often done in a heuristic manner to attain a reasonable visual quality. For FBP there is a filter parameter for each reconstruction channel, and the same holds for the type of regularizer used in the presented MBIR approach resulting in a large number of parameters if they have to be objectively chosen for each channel. The time required to reconstruct a 3D sub-volume comprising 4 slices of size 460×460 from one wavelength bin using the MBIR approach and all the projection views was approximately 1 min (maximum of 250 MBIR iterations) using a machine with 40 cores and 2 Tesla P40 GPUs. Therefore depending on the available computing resources and desired rate at which we seek feedback from the system we can select the appropriate number of slices and wavelength bins to reconstruct over. We note that this compute can be dramatically accelerated with highly optimized MBIR implementations [35,36]; in this paper we focus on demonstrating a proof-of-concept that highlights the quality improvements that can be achieved using the proposed system.

**Figure 4:**
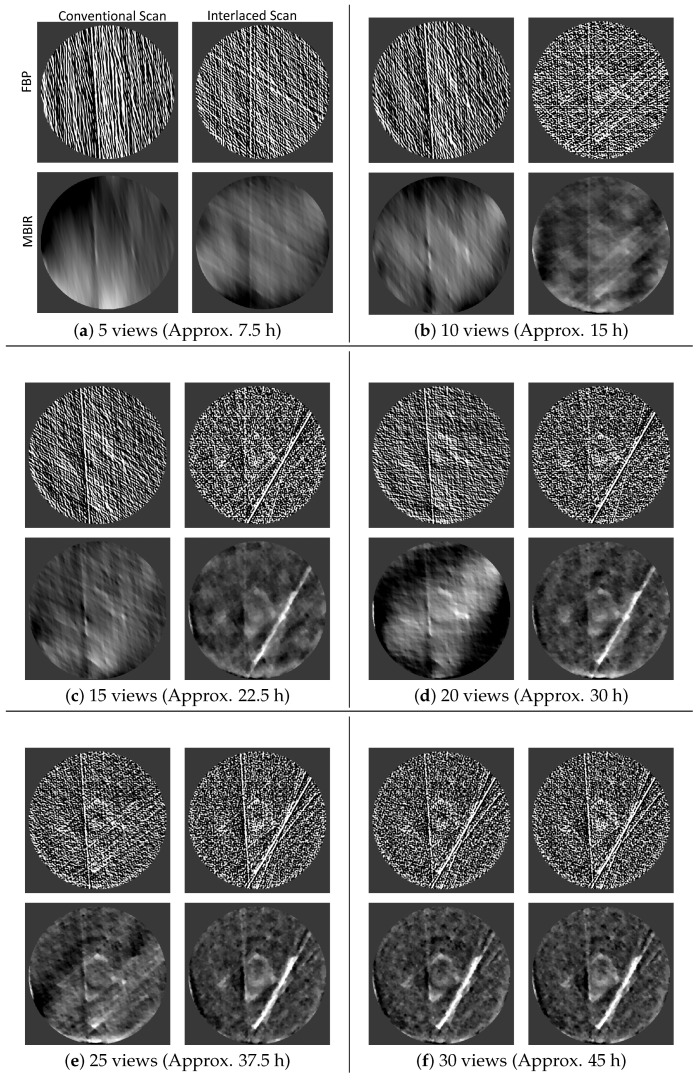
Cross section from the reconstruction of a single wavelength bin using filtered back projection (FBP) and model-based image reconstruction (MBIR) with different scanning protocols. In each inset, the top row is the FBP reconstruction, the bottom is the MBIR. The left column is the result from the conventional scan, while the right column is from the interlaced scanning strategy. Notice that the MBIR reconstructions are of significantly higher visual quality than FBP. Furthermore, the interlaced scanning provides a clearer image of the sample in a more uniform manner thereby providing useful feedback very early in the scan compared to existing protocols. All images are displayed in the viewing window $\left[-0.001,0.0035\right]$.

**Figure 5:**
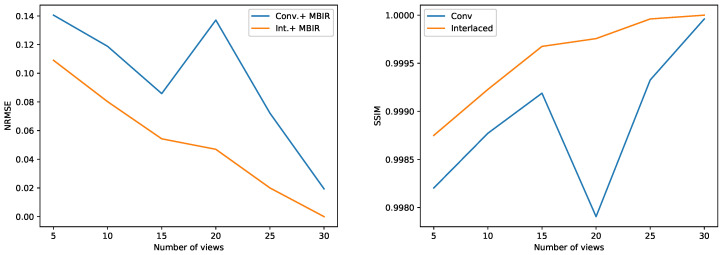
Comparison of normalized root mean squared error (NRMSE) and structural-similarity (SSIM) metrics between the current reconstruction and the final reconstruction in Figure 4 using the MBIR algorithm. Notice that the SSIM and NRMSE converge faster for the interlaced scanning approach compared to the conventional scanning protocol.

Figure 4 show the reconstructions of a single cross section at various stages of the two acquisition protocols when processed using FBP and MBIR algorithms. We emphasize that these results compare reconstructions using different scanning strategies over the $180^{\circ }$ angular range and different reconstruction algorithms corresponding to the acquired data; and not a comparison of how different algorithms perform when we have a sparse projection dataset as might be typical. Notice that even after about 22.5 h of experiment time (15 views), we can start seeing the features of the underlying sample clearly using the MBIR method combined with an interlaced scanning strategy. This illustrates that merely acquiring the data using a standard method combined with a MBIR algorithm is not the best strategy. This result highlights that the interlaced scanning strategy can pave the way for instrument users to be able to view reconstructions in real time and start getting meaningful feedback much prior to the completion of the experiment. This will be crucial for WRNT systems since beam time is at a premium and the making the instrument robust to disruptions can be highly impactful. In Figure 5 we quantify the accuracy of the proposed method by comparing the normalized root mean-squared error (NRMSE) and structural-similarity (SSIM) index [39] of a single cross-section between the current reconstruction and the final reconstruction upon completion of the experiment using a given algorithm. This gives a measure of how similar the “current” reconstruction is to the final solution and how fast it is converging to the desired solution when using the MBIR method but different acquisition protocols. Once again notice that these metrics are consistent with the visual inspection, further highlighting the value of combining an interlaced scan with a MBIR method for WRNT systems.

**Figure 6:**
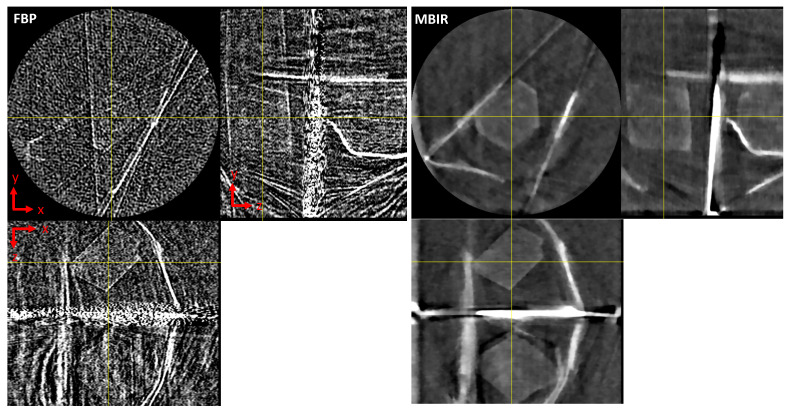
XY, XZ and YZ cross sections of the final reconstruction corresponding to the first wavelength bin. Notice that the features of the sample are clearly visible in MBIR even though the input data has only 30 projections. The filter and regularization parameters are chosen for approximately similar visual resolution. Notice that the MBIR approach results in a significantly lower noise reconstruction compared to the FBP approach.

**Figure 7:**
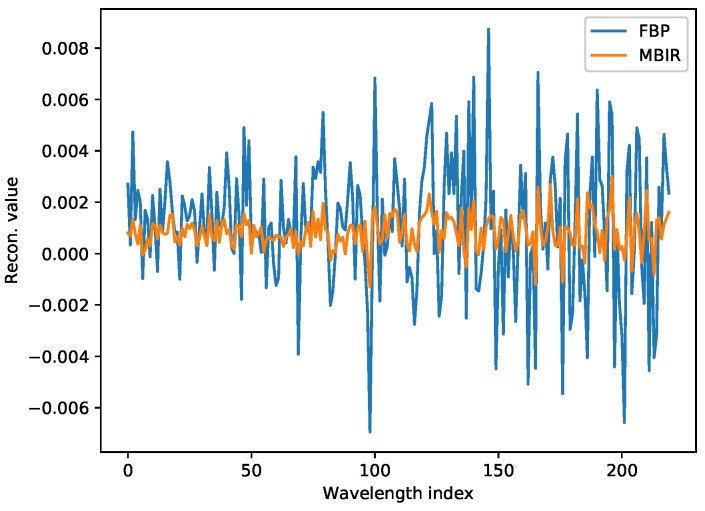
Line profile through the center of the rock. Notice that the FBP produces a very noisy reconstruction as a function of wavelength index.

Figure 6 show the overall qualitative improvements in the final reconstruction between the MBIR and the FBP method for WRNT. Notice that the MBIR method can significantly suppress noise while preserving the resolution of the reconstruction compared to the FBP algorithm. The edges are clearly reconstructed and even the region of the cadmium plate which is highly attenuating is clearly reconstructed compared to the FBP algorithm. Furthermore, a plot of the average reconstructed value in a central patch across the wavelength dimension (Figure 7) shows the significant reduction in noise that can be obtained using the proposed MBIR technique. We believe that these results can be further improved by the use of more sophisticated regularization techniques that exploit correlations across adjacent wavelength bins. Finally, we note that the minor difference in qualitative and quantitative results of MBIR using all the data but in the conventional scanning mode and interlaced mode is due to the effect of the strip-removal filter in TomoPy, whose output varies according to the ordering of the input views.

## 4. Conclusions

In this paper we presented a new method for acquisition and reconstruction for wavelength-resolved neutron tomography beam lines. The core idea behind our approach is to combine an interlaced scanning protocol with the use of a model-based reconstruction algorithm that can produce high-quality reconstructions from low SNR, sparse and incomplete datasets. Our method is ideal for step-and-shoot type systems where the time to acquire the data is significantly higher than the time to position the sample in a certain orientation. An advantage of our approach is that the combination of a new acquisition protocol combined with a model-based reconstruction algorithm can produce high-quality reconstructions even during the course of the experiment; making it more robust to potential disruptions in the experiment and also enabling users to visualize their sample in real-time paving the way for them to modify/terminate the experiment based on this feedback. While this method is ideal for samples with unknown shapes and distribution of features, in the next phase of research we foresee designing sample-adaptive scanning protocols that can enable even more efficient acquisition of the data along the lines of [40].

## Data Availability

The data presented in this study is available on request from the corresponding author.
